# Ocular manifestations of Clade Ib mpox in four South-Kivu health zones, Democratic Republic of the Congo

**DOI:** 10.1371/journal.pgph.0005971

**Published:** 2026-02-18

**Authors:** Deogratias Basedeke Ngoma, Irène Nsimire Ntererwa, Aristote Biringanine Irenge, Marcel Changa Mweze, Patrick de Marie Chimusa Katoto, David Mwenebitu Lupande, Joseph Nelson Siewe Fodjo, Piet Noë, Juliet Sengeri Otiti, Theophile Barhwamire Kabesha, Robert Colebunders, Christophe Van Dijck, Laurens Liesenborghs, Placide Mbala-Kingebeni, Steven Yeh, Jean-Claude Mwanza

**Affiliations:** 1 Department of Ophthalmology, Bukavu Provincial Referral Hospital, Bukavu, Democratic Republic of the Congo; 2 Faculty of Medicine, Catholic University of Bukavu, Bukavu, Democratic Republic of the Congo; 3 Public Health Department, Official University of Bukavu, Bukavu, Democratic Republic of the Congo; 4 National Eye Health and Vision Program, Bukavu, Democratic Republic of the Congo; 5 University of Stellenbosch, Capetown, South of Africa; 6 Center for Tropical Diseases and Global Health, Catholic University of Bukavu, Bukavu, Democratic Republic of the Congo; 7 Department of Laboratory, Bukavu Provincial Referral Hospital, Bukavu, Democratic Republic of the Congo; 8 Global Health Institute, University of Antwerp, Antwerp, Belgium; 9 Rwanda Charity Eye Hospital, Muhanga, Rwanda; 10 Department of Ophthalmology, College of Health Sciences, Makerere University, Kampala, Uganda; 11 Department of Ophthalmology, Official University of Bukavu, Bukavu, Democratic Republic of the Congo; 12 Department of Clinical Sciences, Institute of Tropical Medicine, Antwerp, Belgium; 13 Department of Microbiology, Immunology and Transplantation, KU Leuven, Leuven, Belgium; 14 National Institute for Biomedical Research (INRB), Kinshasa, Democratic Republic of the Congo; 15 Microbiology Service, School of Medicine, University of Kinshasa, Kinshasa, Democratic Republic of the Congo; 16 Department of Ophthalmology and Visual Sciences, Truhlsen Eye Institute, University of Nebraska Medical Center, Omaha, Nebraska, United States of America; 17 Global Center for Health Security, University of Nebraska Medical Center, Omaha, Nebraska, United States of America; 18 Department of Ophthalmology, University of North Carolina-Chapel Hill, Chapel Hill, North Carolina, United States of America; 19 Department of Ophthalmology, University of Kinshasa, Kinshasa, Democratic Republic of the Congo; University of Colorado Anschutz Medical Campus: University of Colorado - Anschutz Medical Campus, UNITED STATES OF AMERICA

## Abstract

With several international outbreaks ongoing, mpox-related eye disease is increasingly detected, yet data on its prevalence remain limited. We conducted a descriptive cross-sectional study in four health zones in South-Kivu Province, Democratic Republic of the Congo, between November 1, 2024 and January 31, 2025, to describe the clinical features of mpox-related ophthalmic disease in hospitalized patients during the ongoing clade Ib outbreak. Routine ophthalmic examination was performed in laboratory-confirmed hospitalized mpox patients. The assessment included visual acuity measurement, inspection of eyelids and ocular surface with a magnifying glass, portable slit lamp biomicroscopy of the anterior segment, and ophthalmoscopy. We examined a total of 366 patients, of whom 210 (57.4%) were male. The median (IQR) age was 8 (4–16) years, 260 (71%) of the patients were younger than 15, and 168 (45.8%) were under 5. Ocular symptoms and disease were recorded in 190 (51.9%) patients. The most common ocular symptom was redness (51.9%), followed by pain (20.0%), itching (18.3%), and conjunctival discharge (16.7%). Conjunctivitis was observed in 113 (30.9%) patients. Other manifestations included blepharoconjunctivitis (7.4%), blepharitis (5.2%), and keratoconjunctivitis (3.3%). Ulcerative and non-ulcerative keratitis were present in 2.5% and 2.2% of the patients, respectively. Two (0.6%) HIV-positive patients had herpes zoster ophthalmicus. Visual impairment was recorded in 5.7% and blindness in 2.5% of the patients. Ophthalmologic manifestations were common during hospitalization in this group of patients. The early occurrence of ophthalmic manifestations requires early ophthalmic assessment for timely diagnosis and treatment to achieve better outcomes. Outbreak frontline healthcare workers should be alert of ocular symptoms such as redness, pain, sensitivity to light, and tearing in both suspected and laboratory-confirmed cases and promptly initiate an ophthalmic evaluation.

## Introduction

Mpox is an emerging viral disease caused by the mpox virus (MPXV), a double-stranded DNA virus that belongs to the Orthopoxvirus genus of the Poxviridae family. To date, two genetically distinct MPXV variants have been described: clade I (subdivided into Ia and Ib) originated in Central Africa and is associated with more severe disease, and clade II (subdivided into IIa and IIb) [1]. In recent years, several worldwide outbreaks have been driven by sustained human-to-human transmission of MPXV. In 2022, clade IIb caused a global outbreak with more than 100,000 reported cases worldwide. In 2024, the emergence of clade Ib in South-Kivu, Democratic Republic of the Congo (DRC), led to a large multi-country outbreak, primarily affecting the African continent but with imported cases reported in several other regions [2].

Following a median incubation period of 13.5 (9.5-19) days [3], clade Ib MPXV infection typically presents with fever, headache, myalgia, sore throat, lymphadenopathy, and a characteristic rash. In addition to bacterial superinfection, pregnancy loss, urogenital complications, and sepsis, ocular manifestations represent an important complication of MPXV infection, sometimes resulting in severe complications and visual impairment. The epidemiology and clinical profiles of mpox-related ophthalmic disease (MPXROD) have been described in series from previous outbreaks [4]. Ophthalmic disease described include periocular lesions (i.e., lesions and inflammation of the lids), conjunctivitis, keratitis, uveitis, scleritis, and periorbital lesions. Serious complications include bacterial superinfection, corneal scarring, necrotizing scleritis, corneal perforation, and phthisis bulbi. The prevalence of MPXROD appears to vary by clade, with lower rates in clade II- [5–9] than clade I-affected patients [10–14].

In August 2024, the continued rapid rising numbers of confirmed mpox cases, particularly in eastern DRC, led the WHO and Africa CDC to develop a Joint Continental Mpox Plan centered on ten key pillars, which included the development of a research roadmap for mpox. They appealed to the international community for concerted efforts to conduct research to help understand all aspects of the disease and contribute to the control of the outbreak [15]. Given the lack of data on the prevalence, incidence, and clinical spectrum of MPXROD, we designed this study to provide information that will fill the gap in knowledge on the prevalence and the clinical profile of clade Ib-related MPXROD among hospitalized mpox patients in four health zones in South-Kivu province within the DRC. Understanding the spectrum of ocular mpox during the active phase of clade Ib MPXV infection is paramount for vision health, quality of life, and public health.

## Materials and methods

### Ethics statement

The study received approval from the Institutional Health Ethics Committee of the Institut Supérieur des Techniques Médicales (ISTM) of Bukavu (Approval number ISTM-BKV/CRPS/CIES/MLM/019/2024). Its execution complied with the tenets of the Declaration of Helsinki and a written informed consent to participate in the study (including to acquire and publish pictures of eye lesions if needed) was obtained from all adult and parents of minor (<18 years) participants. To maintain confidentiality of individual participants, the data were de-identified.

### Geographical context

**Fig 1:**
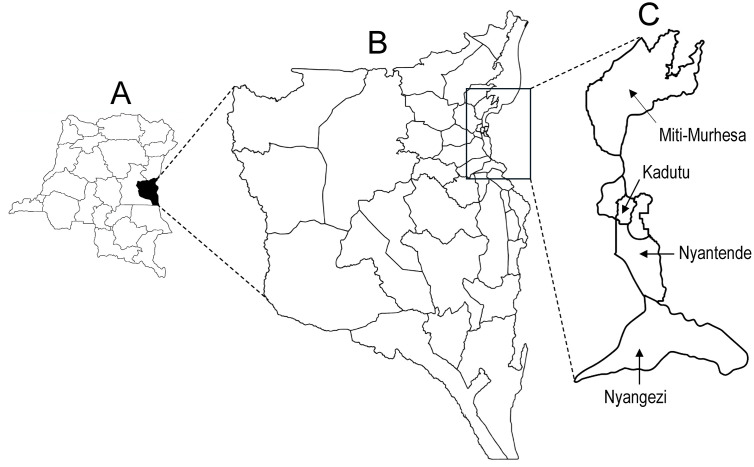
Map depicting the 26 provinces of the DRC, with South-Kivu highlighted in black (A), map of South-Kivu with its 36 health zones (B), and the 4 zones where the study took place (C).

South-Kivu is one of the 26 DRC provinces, located in the eastern part of the country, south of North-Kivu province (Fig 1). The province is subdivided into 34 health zones, including Miti-Murhesa, Nyangezi, Nyantende, and Kadutu, which were the sites of this study (Fig 1). Nyantende shares a border with Rwanda whereas Nyangezi shares borders with both Rwanda and Burundi.

### Epidemiological context

South-Kivu province is one of the epicenters of the current mpox outbreak within the DRC, and the province from which the subclade Ib emerged in 2023 before spreading from the epicenter of Kamituga health zone to other South-Kivu health zones, provinces and neighboring countries [16,17]. Miti-Murhesa has been a major hotspot for the ongoing mpox outbreak, recording a high number of cases, particularly among children with 3,790 cases and 17 deaths reported by the end of October 2024, making it one of the most heavily affected areas. By the end of 2024 at least 4,700 cases were confirmed in Miti-Murhesa, representing a substantial proportion of all cases in South-Kivu. Nyangezi, Kadutu, and Nyantende were also among the most affected areas following a surge in the number of cases by June 2024. Miti-Murhesa has two mpox treatment centers (MTC) whereas each of the other three health zones has one treatment center per zone, where suspected mpox cases are triaged and laboratory-confirmed cases are isolated and treated until discharge.

### Study design and patient population

We conducted a cross-sectional descriptive, prospective study from November 1, 2024 to January 31, 2025. We included in the study subjects who had been diagnosed with mpox that was laboratory-confirmed by either GeneXpert or RT-PCR and hospitalized in an MTC at the time of the ophthalmologic assessment. We excluded those who refused to participate in the study and/or were unable to undergo ophthalmic examination due to poor general health. GeneXpert testing (most subjects) was performed at the Provincial Health Division of South-Kivu whereas RT-PCR was performed only in a few participants at the Institut National de Recherche Biomédicale (INRB) in Goma (North-Kivu) due to a temporary malfunction of the GeneXpert system. MPXV sequencing was not performed. HIV status was assessed with a rapid diagnostic test. Due to resource limitations, comprehensive diagnostic testing for other potential coinfections that may present with overlapping clinical features, such as varicella zoster virus (VZV), herpes simplex virus (HSV), was not performed. Demographic characteristics (i.e., age and sex) and ocular symptoms including redness, pain, foreign body sensation, tearing, light sensitivity, and blurry vision were recorded.

### Ophthalmic examination

A certified ophthalmologist performed a routine ophthalmic examination at the patient’s bedside within the MTC. It notably included measurement of uncorrected visual acuity or corrected with spectacles at 2 meters, visual inspection of the periocular region, and careful examination of the eyelid margins and lashes, conjunctiva, and cornea through a 10x magnifying glass. We used a portable slit-lamp to examine the ocular surface and anterior segment of the eye. The ocular fundus was examined under pupil dilation. Corneal staining with topical fluorescein was performed in all cases of conjunctivitis and keratitis. Intraocular pressure was deferred. We performed all assessments under strict adherence to recommended infection prevention and control guidelines [18], with the examinators in personal protective equipment including single-use nasal mask, vinyl gloves, gown, and head and shoe covers. MPXROD was defined as any condition affecting the eye and attributable to MPXV, including eyelid, periocular and orbital lesions, conjunctivitis, keratitis, scleritis, and uveitis. Visual impairment and blindness were defined per the WHO classification.

### Statistical analysis

Categorical variables were expressed as frequencies and/or percentages, whereas quantitative variables were represented as medians with interquartile ranges (IQR). The Mann-Whitney U test was used to compare medians of quantitative variables (i.e., age, visual acuity) between those with vs. without ocular disease. A Pearson Chi-square test was used to compare proportions of patients between mpox patients with and without disease. The analyses were performed using SPSS 27.0 (IBM, Armonk, New York, NY, USA). For all comparisons, statistically significant difference was set at a p-value < 0.05.

## Results

### General characteristics of mpox patients

We included a total of 366 mpox patients whose general characteristics are provided in Table 1. The overall median age (interquartile range, IQR) of 9 (4–15) years, without significant difference between patients with and those without ocular disease (p = 0.67). Most participants (73.2%) were younger than 15 years of age, and the distribution of age groups (<15 vs. ≥ 15 years) was comparable between those with and without ocular disease (p = 0.35). Male participants accounted for 57.4% of all patients, and the sex distribution did not differ significantly between groups (p = 0.83). Patients from Miti-Murhesa health zone accounted for 45.9% of all participants. The proportions of patients with and without ocular disease were similar across all health zones (p = 0.51). Although mpox vaccination was briefly introduced in the study area, none of the enrolled patients received the vaccine due to severely limited vaccine availability and vaccine refusal among a subset of individuals. Only three patients (45, 47, and 53 years old) had scars from smallpox vaccination.

**Table 1:** General characteristics of all mpox patients.

Parameter	All (n = 366)	Ocular Disease (n = 190)	No Ocular Disease (n = 176)	p
Sex				
Male, n (%)	210 (57.4)	110 (57.9)	100 (56.8)	0.83
Female, n (%)	156 (42.6)	80 (42.1)	76 (43.2)	
Median age (IQR)	9 (4-15)	8 (5-15)	9 (4-15)	0.67
Age groups, n (%)				
< 15 years	260 (71.0)	139 (73.2)	121 (68.8)	0.35
≥ 15 years	106 (29.0)	51 (26.8)	55 (31.2)	
Health districts, n (%)				
Miti-Murhesa	168 (45.9)	83 (43.7)	85 (48.3)	0.51
Nyangezi	112 (30.6)	57 (30.0)	55 (31.3)	
Nyantende	71 (19.4)	40 (21.1)	31 (17.6)	
Kadutu	15 (4.1)	10 (5.2)	5 (2.8)	
HIV-positive	2 (0.55)	2 (1.1)	0 (0)	0.16

### Ocular symptoms and ocular disease

The profile and frequency of ocular symptoms and ocular disease among 366 mpox inpatients are detailed in Table 2. Ocular symptoms and disease were observed in 190 (51.9%) of the patients. The most common ocular symptoms were redness (51.9%), pain (20.0%), itching (18.3%), and conjunctival discharge (16.7%). Conjunctivitis, present in 30.9% of all patients, was the most frequent ocular disease, followed by blepharoconjunctivitis (7.4%), isolated blepharitis (5.2%), and keratoconjunctivitis (3.3%). Non-ulcerative and ulcerative keratitis were observed in 9 (2.2%) and 8 (2.5%) patients, respectively. Unilateral or bilateral visual impairment occurred in 21 (5.7%) patients, and 9 (2.5%) patients were classified as blind. All cases of blindness were due to corneal ulcerations.

**Table 2:** Ocular manifestations in 366 mpox patients.

Parameter	N (%)
Symptoms/ signs	
Eye redness	190 (51.9)
Eye pain	73 (20.0)
Eye itching	67 (18.3)
Conjunctival discharge	61 (16.7)
Photophobia	54 (14.8)
Foreign body sensation	22 (11.6)
Tearing	9 (4.7)
Blurred vision	6 (3.2)
Ocular disease	
Conjunctivitis	113 (30.9)
Blepharoconjunctivitis	27 (7.4)
Blepharitis	19 (5.2)
Keratoconjunctivitis	12 (3.3)
Ulcerative keratitis	9 (2.5)
Non-ulcerative keratitis	8 (2.2)
Herpes zoster ophthalmicus	2 (0.6)
Vision status	
Normal	336 (91.8)
Visual impairment	21 (5.7)
Blindness	9 (2.5)

**Fig 2:**
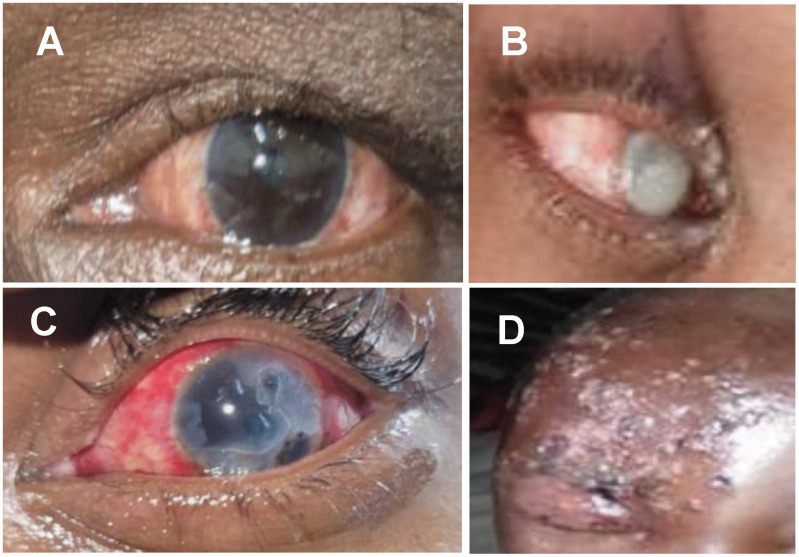
Selected four examples of mpox patients with ocular disease showing a the left eye of a patient with mild bilateral conjunctivitis and 6/6 vision (A), the right eye of a child with bilateral blinding corneal ulcer covering the entire cornea and vision limited to light perception (B); the left eye of a patient with unilateral diffuse conjunctival hyperemia with peripheral keratitis progressing toward the center of the cornea, with visual acuity reduced to 6/36 (C), and an HIV-positive child with a right-side herpes zoster ophthalmicus and 6/24 vision in the right eye (D).

Representative cases of conjunctivitis and corneal ulcers are shown in Fig 2. Herpes zoster ophthalmicus was observed in 2 (0.6%) HIV-positive patients, a 10-year-old child and a 21-year-old female soldier, both under treatment (CD4 counts not available). The diagnosis of herpes zoster ophthalmicus was made clinically based on the presence of a unilateral rash in the trigeminal nerve’s ophthalmic (V1) dermatome. They both had ipsilateral eyelid edema and conjunctivitis.

## Discussion

In this study we describe the ocular manifestations observed among mpox patients during hospitalization. Although the current clade Ib mpox outbreak in the DRC was declared a PHEIC and a PHECS in August 2024 by the WHO and Africa CDC, respectively, data on the ophthalmic findings associated with this MPXV variant remain lacking. This study focuses on ocular manifestations observed in patients with mpox during stay in MTCs in four health zones in South-Kivu, where clade Ib mpox predominates. Our findings provide timely evidence that ocular symptoms and signs are common, affecting approximately half of all patients, with about 5% experiencing visual impairment.

It has been demonstrated that clade Ib is the dominant circulating MPXV variant in South-Kivu, with sexual contact being the primary transmission mode, and secondary transmission by close contact within households [19]. Since most of our patients were children, it is likely that they became infected through household or community transmission via close physical contact. Notably, 71% of all participants and 73.2% of those with ocular disease were under the age of 15 years (median age: 5 years). This finding is concerning and aligns with recent reports, indicating that pediatric patients represent a sizable proportion of mpox patients within the DRC [20,21] and that younger age is associated with higher risk for severe disease, including ocular involvement, as well as higher fatality rates [21]. We recently reviewed data collected in 216 mpox patients from 2007 to 2011 in Kole (Sankuru District, Kasai Oriental Province, DRC), where mpox has been associated with subclade Ia, and reported that most patients with ocular manifestations were children (median age: 5.7 years) [22]. Pediatric patients are particularly prone to eye rubbing, which increases the risk of self-inoculation of MPXV from cutaneous lesions to the ocular surface, as well as the risk of secondary bacterial infection and subsequent corneal ulceration. Due to the higher risk of permanent visual loss, along with differences in clinical presentations, disease course, monitoring and follow-up, and therapeutic considerations and drug safety profiles, the management of ocular mpox should differ between pediatric and adult patients. While the foundational management of ocular mpox is similar in adults and children, pediatric care should be more proactive, closely monitored, and prevention-focused, with early involvement of eye care professionals. These distinctions are clinically important and should be reflected in outbreak response guidelines to minimize avoidable, lifelong visual disability in children.

The prevalence of MPXROD varies considerably across mpox outbreaks, geographic regions, and MPXV clades. The prevalence of ocular disease in our study was 51.9%, which is higher than previously reported in the DRC: 4.3% across several regions [10], 20.7-24.1% in Tshuapa province [11,13,14], 8.3% in Sankuru province [22], and 7.4% among mpox patients examined in Kole between 2007 and 2011 [23]. It is noteworthy that prior studies relied on general clinical examinations performed by non-ophthalmologists, often in resource-limited settings. Consequently, subtle or early ocular manifestations, such as mild conjunctivitis, punctate keratitis, or eyelid margin involvement, may have been underrecognized or misclassified. In contrast, our study benefited from systematic ophthalmic evaluation by a team of ophthalmologists, which increased the sensitivity for detecting mild forms of ocular disease. This difference likely contributed to underestimation of MPXROD prevalence in earlier reports within the DRC. Thus, our findings suggest that ocular involvement in mpox patients may be more common than previously reported, particularly when actively sought through specialized evaluation. During the 2022 outbreak, which was caused by clade IIb, rates of MPXROD were 15% in a nationwide study in Nigeria [24] and 0.3-3.8% across Europe [6,8,9,25,26]. In Latin America, nationwide studies reported rates were 0.9% in Mexico [27] and 1% in Brazil [7]. Studies in North America reported a prevalence of 0.3% in Canada [28] and 6.2% among cases reported to the New York City (USA) Department of Health and Mental Hygiene surveillance system [29]. In Asia, a multicenter study conducted in South Korea found that 1.7% of mpox patients had ocular disease [30]. Thus, the rate of ocular disease observed in this study area of the DRC, where clade Ib MPXV predominates, appears higher than that reported in regions outside the DRC where clade II MPXV circulates. Although multiple factors may contribute to this difference, the predominance of clade Ib during the current outbreak in eastern DRC, associated with more severe disease, may partially account for the observed trend [17]. However, confirmation across other areas of the South-Kivu province is essential to determine whether this pattern is reproducible. Consistent observations of increased ocular involvement in clade Ib infections, using standardized ocular definitions and comparable case ascertainment, would strengthen the inference of a clade Ib-specific effect. Conversely, heterogeneity in findings would suggest that host factors, differences in access to care, timing of presentation, HIV prevalence, rates of secondary infections, and clinical awareness of ocular complications could influence the observed burden and severity of eye disease more than viral clade alone. This underscores the need for coordinated surveillance, integration of ophthalmologists into mpox care teams to ensure timely and accurate diagnosis, appropriate management of potentially vision-threatening complications, and viral sequencing linked to clinical phenotypes across the DRC.

The most common presentation of MPXROD in this study population was conjunctivitis, affecting one in three patients. A recent study among hospitalized mpox patients during the current clade Ib outbreak in Kamituga, South-Kivu province, reported conjunctivitis in 25.5% of patients [31]. A comparison of the clinical characteristics of mpox across the DRC yielded pooled prevalences of conjunctivitis of 10.4% in two newly affected provinces (Kinshasa and South-Kivu) and 28.3% in five endemic provinces (Equateur, Mai-Ndombe, South-Ubangi, Tshopo, and Tshuapa) [12]. In earlier series from the DRC, conjunctivitis was described in 15.2%-24.1% of mpox patients and was consistently the leading ocular disease among infected patients [10,11,13,32]. In Nigeria, where mpox was caused by clade IIb, conjunctivitis was diagnosed in 26% of the patients during the 2017–2018 and in 15% of the patients during the 2022 mpox outbreak [24,33]. In contrast, most studies outside Africa have reported conjunctivitis in 0.3% to 2.6% of mpox patients [7–9,26,28,30,34–39]. The predominance of conjunctivitis as the most frequently reported MPXROD likely reflects both viral tropism and exposure dynamics at the ocular surface. Autoinoculation from eyelid, facial, or periocular lesions provides a plausible and efficient route of transmission, as contaminated hands can directly introduce virus onto the conjunctival epithelium. This risk is amplified by the high burden of facial and eyelid lesions in mpox, which are often pruritic or painful and therefore encourage frequent touching. In many settings, limited patient awareness that eye rubbing should be avoided, particularly during active skin disease, further increases the likelihood of self-inoculation. Together, these factors help explain why conjunctivitis predominates over deeper ocular tissues involvement and highlight the importance of early counseling on hand hygiene and avoidance of eye contact as simple, preventive measures to reduce ocular complications. Additional factors that may contribute to the high frequency of conjunctivitis in mpox beyond autoinoculation are: (1) Mucosal tropism of Orthopoxviruses. Orthopoxviruses have a known capacity to infect mucosal epithelium. Conjunctival involvement may therefore represent part of a broader mucocutaneous disease spectrum rather than purely mechanical spread. (2) Conjunctival anatomical and immunologic vulnerability. The conjunctiva is more susceptible to initial viral replication and inflammation because it is an openly exposed mucosal surface with limited innate antiviral defenses compared with the cornea. (3) Viral spread via hematogenous and/or lymphatic pathways. During viremia, the virus may reach the conjunctiva via systemic dissemination, causing conjunctivitis even in the absence of obvious periocular or skin lesions. (4) Tear film contamination. The tear film may be contaminated by viral particles shed from facial (eyelids) lesions through blinking or drainage in the nasolacrimal system, ultimately facilitating conjunctival exposure without autoinoculation through eye rubbing. (5) Secondary inflammation rather than direct infection. In some cases, conjunctivitis may reflect immune-mediated inflammation triggered by systemic immune activation or adjacent skin lesions, rather than primary conjunctival infection. (6) Under-recognition of subtle conjunctivitis. Mild conjunctival injection or discharge is more easily detected and reported than deeper ocular pathology, potentially inflating its apparent prevalence relative to other ocular manifestations. (7) Coexisting infections or irritation. Bacterial superinfection, viral co-pathogens, or environmental irritants in resource-limited or crowded settings may exacerbate or unmask conjunctival inflammation in patients with mpox. Together, these mechanisms suggest that conjunctivitis in mpox is likely multifactorial, arising from a combination of direct viral exposure, mucosal susceptibility, systemic spread, and host immune responses.

The proportion of patients with non-ulcerative keratitis was 5.7% in the present study. In two prior studies within the DRC, keratitis was observed in 4.3% and 3.3% of the patients [10,32]. Rates of keratitis from other series vary from as high as 10.6% in one study in Nigeria [24] to lower rates varying between 0.4% and 2.9% outside Africa [6,38,40,41]. In mpox patients, keratitis can lead to more severe complications, including corneal ulcers, scarring, and sometimes irreversible blindness. Other serious complications include corneal perforation and phthisis bulbi. Notably, severe forms of keratitis have been described across the full clinical spectrum of mpox, including forms without obvious skin lesions [42], highlighting the importance of early recognition and consideration of prompt management with both systemic and topical antivirals to prevent vision loss and further transmission. Corneal ulceration was observed in 2.5% of our patients, higher than the 0.5% reported by both Mbala-Kingebeni et al in Kole [22] and Pazos et al in Spain [9], but consistent with 2.6% (one patient) among 38 patients from the DRC studied by Breman et al between 1970 and 1979 [43]. Corneal ulcer appears to be an uncommon event during clade Ib MPXV infection. Since all types of keratitis, including ulcerative types, are sight-threatening complications, patients’ and healthcare workers’ continuous education to improve recognition of early signs and prompt ophthalmology referral is crucial for appropriate diagnosis and management to prevent progression.

Although mpox may lead to partial or complete loss of vision because of severe ocular disease, visual impairment and blindness have been infrequently observed during previous outbreaks. Compared to 0.6% and 1% of mpox patients with visual impairment during the 2022 outbreak reported by Fink et al [26] and Rodriguez-Badillo et al [41], respectively, the rate in our study was 5.7%. Interestingly, Malembi et al recently showed a stark difference in visual impairment rates between newly affected areas (0.2%) during the current mpox outbreak and known endemic areas (6.1%) within the DRC, a contrast hypothetically attributed to virulence differences between clades Ib and Ia [12]. An investigation by Jezek et al in the DRC dated in the mid-1980s found 2.1% of patients with corneal scar-related visual impairment [10]. There were 9 (2.5%) unilaterally or bilaterally blind patients in our cohort of mpox patients. Among the patients described by Breman et al in the DRC, one child (2.6%) went blind in one eye due to corneal scar resulting from ulceration [43]. Corneal scar also caused unilateral blindness in 4 (1.4%) children reported by Jezek et al [10]. Despite the ongoing simultaneous mpox outbreaks due to clade Ia and Ib within the DRC, efforts have been mostly concentrated on the containment of the clade Ib spread. However, it is important not to lose sight of the fact that there is already a staggering number of clade Ia MPXV survivors with mpox-related corneal scars in endemic areas of the DRC, particularly children, who need corneal transplant to restore vision, improve quality of life, and provide hope. Urgent research involving cornea specialists is needed to determine the contribution of mpox to corneal blindness in these endemic areas and the need to support a corneal transplant program.

Compelling data exist on the co-existence of MPXV with other viral agents, including HIV and VZV, creating diagnostic challenges, raising significant concerns about patient outcomes, and requiring precautionary management considerations [44,45]. While several cases of ocular manifestations in mpox patients with HIV have been reported [46–48], we could not find a single report of herpes zoster ophthalmicus in an HIV-positive mpox patient. In people with HIV, the clinical phenotypes of MPXV-, HSV-, and VZV-related ocular disease substantially overlap, rendering clinical diagnosis alone insufficiently specific. We acknowledge that in the absence of virologic testing of ocular specimens due to limited laboratory capacity, attribution of ocular findings to mpox in such patients remains probabilistic rather than definitive and may result in misclassification. This diagnostic uncertainty has direct implications for both the reported prevalence of ocular mpox and for management, particularly with respect to antiviral selection, treatment duration, and infection control measures. This underscores the need for and importance of ocular sample PCR (i.e., multiplex PCR testing) and syndromic, rather than pathogen-exclusive, management algorithms to mitigate misattribution when laboratory confirmation is not feasible.

This study has limitations that should be acknowledged. First, we only examined patients who were hospitalized and isolated in MTCs within an area of the DRC where clade Ib predominates. Thus, the findings of this study may only apply to this setting until corroborated by studies conducted in other areas affected by clade Ib MPXV. Second, this descriptive study does not address an important and clinically relevant question to general health care providers (i.e., nurses, general practitioners), who are often the first point-of-contact for patients with mpox. Although detailed management of ocular lesions was beyond the scope of this study, all patients with ocular findings considered attributable to mpox received treatment guided by clinical assessment. Management approaches were heterogeneous and resource-limited, typically consisting of topical antibiotics (drops or ointment depending on availability at the treatment center) for conjunctivitis and keratitis, suspected secondary infection or superinfection prevention, and lubricants for comfort. Access to topical antivirals or advanced corneal care was not possible. Corticosteroids were not used. No patients received systemic antiviral therapy (e.g., tecovirimat) or topical antivirals (e.g., trifluridine or ganciclovir), as these treatments were not available at the study sites or sold on the local market. Third, follow-up visits and longitudinal assessments of ocular disease were not feasible because the study area became an active armed conflict zone a week after completion of the study. This sudden deterioration in security led to the displacement of both patients and healthcare personnel, precluding planned follow-up evaluations and limiting our ability to characterize the evolution of ocular lesions and their short-term visual consequences. This highlights the operational challenges of conducting clinical research during infectious disease outbreaks in humanitarian and conflict-affected settings. Despite these limitations, the descriptive epidemiology presented here constitutes a critical first step in outbreak settings by quantifying the burden, spectrum, and severity of ocular involvement among hospitalized patients with clade Ib mpox. The observation that more than half of patients experienced ocular symptoms, with nearly 5% developing keratitis and 2.5% progressing to blindness secondary to corneal ulceration, reveals a substantial and previously underrecognized risk of vision-threatening disease. These findings have immediate clinical relevance, underscoring the need for early ocular screening, timely referral, and implementation of preventive eye care strategies within MTCs. To better characterize the epidemiology of ocular mpox, our cross-sectional findings will need to be complemented by longitudinal studies designed to define the natural history of ocular involvement, distinguish transient from progressive disease, evaluate virologic clearance at the ocular surface, and determine predictors of both ocular disease and adverse visual outcomes. Such studies would also integrate standardized ophthalmic management protocols and outcomes to allow assessment of treatment effectiveness, optimal timing of ophthalmologic referral, and the durability of visual recovery following acute infection.

## Conclusions

The findings of this study provide information on the clinical profile of ocular mpox among hospitalized patients with clade Ib MPXV in this study area, and can be summarized as follows: (1) Ocular mpox during hospitalization primarily involves the ocular surface, with conjunctivitis as the most common manifestation; (2) The high frequency of ocular disease supports early ophthalmologic evaluation prior to discharge, as well as targeted training of frontline healthcare workers to recognize and promptly refer evolving ocular symptoms; (3) In areas affected by the clade Ib outbreak in eastern DRC, mpox should be included in the differential diagnosis of conjunctivitis and keratitis; and (4) The outbreak exposes critical gaps in eye-care infrastructure in remote settings, underscoring the need to strengthen vision health systems and implement standardized assessment and management protocols to prevent avoidable vision loss in future mpox outbreaks.

## Supporting information

S1 Data Dataset used for the analysis of clade Ib MPXV-related ocular manifestations in four South-Kivu health zones, Democratic Republic of the Congo. (XLS)
